# Mapping Helminth Co-Infection and Co-Intensity: Geostatistical Prediction in Ghana

**DOI:** 10.1371/journal.pntd.0001200

**Published:** 2011-06-07

**Authors:** Ricardo J. Soares Magalhães, Nana-Kwadwo Biritwum, John O. Gyapong, Simon Brooker, Yaobi Zhang, Lynsey Blair, Alan Fenwick, Archie C. A. Clements

**Affiliations:** 1 School of Population Health, University of Queensland, Herston, Queensland, Australia; 2 Neglected Tropical Diseases Control Programme, Ghana Health Service, Accra, Ghana; 3 Research and Development Division, Ghana Health Service, Accra, Ghana; 4 School of Public Health, College of Health Sciences, University of Ghana, Legon, Accra, Ghana; 5 London School of Hygiene and Tropical Medicine, London, United Kingdom; 6 Malaria Public Health and Epidemiology Group, KEMRI/Wellcome Trust Collaborative Programme, Nairobi, Kenya; 7 Regional Office for Africa, Helen Keller International, Dakar, Senegal; 8 Department of Infectious Disease Epidemiology, Imperial College London, London, United Kingdom; 9 Australian Centre for International and Tropical Health, Queensland Institute of Medical Research, Herston, Queensland, Australia; Imperial College London, Faculty of Medicine, School of Public Health, United Kingdom

## Abstract

**Background:**

Morbidity due to *Schistosoma haematobium* and hookworm infections is marked in those with intense co-infections by these parasites. The development of a spatial predictive decision-support tool is crucial for targeting the delivery of integrated mass drug administration (MDA) to those most in need. We investigated the co-distribution of *S. haematobium* and hookworm infection, plus the spatial overlap of infection intensity of both parasites, in Ghana. The aim was to produce maps to assist the planning and evaluation of national parasitic disease control programs.

**Methodology/Principal Findings:**

A national cross-sectional school-based parasitological survey was conducted in Ghana in 2008, using standardized sampling and parasitological methods. Bayesian geostatistical models were built, including a multinomial regression model for *S. haematobium* and hookworm mono- and co-infections and zero-inflated Poisson regression models for *S. haematobium* and hookworm infection intensity as measured by egg counts in urine and stool respectively. The resulting infection intensity maps were overlaid to determine the extent of geographical overlap of *S. haematobium* and hookworm infection intensity. In Ghana, prevalence of *S. haematobium* mono-infection was 14.4%, hookworm mono-infection was 3.2%, and *S. haematobium* and hookworm co-infection was 0.7%. Distance to water bodies was negatively associated with *S. haematobium* and hookworm co-infections, hookworm mono-infections and *S. haematobium* infection intensity. Land surface temperature was positively associated with hookworm mono-infections and *S. haematobium* infection intensity. While high-risk (prevalence >10–20%) of co-infection was predicted in an area around Lake Volta, co-intensity was predicted to be highest in foci within that area.

**Conclusions/Significance:**

Our approach, based on the combination of co-infection and co-intensity maps allows the identification of communities at increased risk of severe morbidity and environmental contamination and provides a platform to evaluate progress of control efforts.

## Author Summary

Urinary schistosomiasis and hookworm infections cause considerable morbidity in school age children in West Africa. Severe morbidity is predominantly observed in individuals infected with both parasite types and, in particular, with heavy infections. We investigated for the first time the distribution of *S. haematobium* and hookworm co-infections and distribution of co-intensity of these parasites in Ghana. Bayesian geostatistical models were developed to generate a national co-infection map and national intensity maps for each parasite, using data on *S. haematobium* and hookworm prevalence and egg concentration (expressed as eggs per 10 mL of urine for *S. haematobium* and expressed as eggs per gram of faeces for hookworm), collected during a pre-intervention baseline survey in Ghana, 2008. In contrast with previous findings from the East Africa region, we found that both *S. haematobium* and hookworm infections are highly focal, resulting in small, localized clusters of co-infection and areas of high co-intensity. Overlaying on a single map the co-infection and the intensity of multiple parasite infections allows identification of areas where parasite environmental contamination and morbidity are at its highest, while providing an evidence base for the assessment of the progress of successive rounds of mass drug administration (MDA) in integrated parasitic disease control programs.

## Introduction

Parasitic infections caused by *Schistosoma haematobium* (the aetiological agent of urinary schistosomiasis) and hookworm (a soil-transmitted helminth; STH) are widely endemic among human populations in sub-Saharan Africa (SSA) [1],[2]. The geographical distribution of these infections is known to be driven by environmental and climatic factors that influence parasite populations and those of the snail intermediate host of schistosomes [3]. Additionally, socioeconomic inequalities in human populations at risk, particularly in access to clean water and sanitation, housing, and the access to treatment impact on the observed distribution of these parasitic infections [2], [4]. Control efforts rely on accurate geographical identification and enumeration of populations most at risk of morbidity (i.e. co-infected and/or with intense infections) [5], [6]. Morbidity, including iron-deficiency anaemia, reduced growth and impaired cognition, is exacerbated by multiple species infections (co-infection) and high parasite burden (i.e. high infection intensity) [7]. Although co-infection and infection intensity are the indirect morbidity indicators most sensitive to changes in parasite transmission, contemporary control programs based on mass drug administration (MDA) are planned according to the identification of communities above established single-species prevalence of infection thresholds [8], [9].

The number of adult worms is particularly difficult to measure and a proxy for infection intensity is often used such as the egg concentration in urine (in the case of *S. haematobium*) or in stool (in the case of intestinal schistosomiasis and STHs). The number of eggs that are passed in the urine or stool is determined by important non-linearities in worm life-cycles such as fecundity of female worms and density-dependent development [10], [11]. In endemic populations, the occurrence of infections that lead to high egg output determines the level of environmental contamination which partly contributes to transmission. Therefore, targeting treatment delivery to communities with a high proportion of co-infected and/or to those with high egg output could lead to more efficient reduction of transmission and severe morbidity compared to targeting treatment based on prevalence of single infections.

With the aim of assisting the planning and implementation of MDA, model-based geostatistics (MBG) has been used to produce predictive empirical maps of prevalence of infection at different spatial scales [12]–[16]. The MBG approach provides an extensive set of spatial modeling tools for assessing the geographical overlap of multiple parasite infections [17]. One approach is overlaying prevalence of infection maps for multiple parasites (i.e. co-endemicity mapping) [18]; alternatively, spatial multinomial models can be used to predict the prevalence of mono- and co-infection [3], [19]. Recently, Brooker et al. [3] have mapped *S. mansoni* and hookworm mono- and co-infection in the East African region; thus far no studies have been reported at the national or regional scale in West Africa.

Examples of MBG studies that have analysed the spatial distribution of single-species intensity of infection are available in the literature [13], [20], [21]. We have recently suggested extending this approach to the overlay of predictive maps of intensity of infection (i.e. mapping co-intensity) to allow the identification of common areas of high transmission of multiple parasite species where integrated treatment could be prioritized [17]. To date there are no reported studies in the literature that have mapped co-intensity profiles.

Recently, with financial and technical support from the Schistosomiasis Control Initiative (SCI), three contiguous countries in the Sahelian zone of West Africa (Burkina Faso, Mali and Niger) conducted coordinated national cross-sectional school-based parasitological surveys [22]. Based on these surveys, Bayesian geostatistical analyses were conducted for estimating the geographical distribution of *S. haematobium* and *S. mansoni* infection metrics in these countries [13], [14], [23]. An important country in the region with respect to helminth transmission is Ghana. The construction of the Akosombo dam from 1962 to 1967, created a vast area now known as Lake Volta (approximately 8,500 km^2^) suitable for the breeding of freshwater snails that serve as intermediate hosts of schistosomiasis. Since then, schistosomiasis in Ghana assumed major importance as a public health problem in the country, and the prevalence of *S. haematobium* rose from 5–10% before the construction of the dam to >90% in most communities along lake Volta [24]–[28]. Similarly, the construction of numerous agricultural dams throughout the Upper East Region between 1958 and 1964 resulted in a rise in prevalence of *S. haematobium* from 17% to 51% [29]. Studies in children 15–19 years of age in the North Western Region [30] and in the Southern Region of Ghana [31], [32], [33] have yielded prevalence estimates of 34% and 60–83.9%, respectively. In a study in infants (1–5 years of age) in the Central region the prevalence of urinary schistosomiasis was estimated to be 11% [34].

Prior to the commencement of the Ghana Health Service Neglected Tropical Disease (GHS NTD) program in five regions (Upper West, Upper East, Northern, Western, and Central) in 2008, national teams collected data for the national mapping of schistosomiasis and STHs with technical assistance of SCI. Before the inception of the GHS NTD program, limited effort had been carried out in a few areas around Lake Volta and no sizeable MDA of schistosomiasis had been implemented in the country. Incidentally for STHs, the Global Program to Eliminate Lymphatic Filariasis (GPELF) was ongoing in lymphatic filariasis (LF)-endemic areas (mainly in the west and north of the country), distributing ivermectin plus albendazole. Therefore the data collected truly represent the burden of schistosomiasis and STH infections before the implementation of the nationwide program.

In this paper, we describe data from the 2008 pre-intervention national helminth survey in Ghana and predict for the first time the prevalence of *S. haematobium* and hookworm mono- and co-infections and intensity of infection, as measured by egg counts, in Ghana. We hypothesize that the combined use of co-infection and co-intensity maps has the potential to further broaden the range of spatial predictive decision-support tools to aid targeting the delivery of integrated MDA and may constitute an important cartographic resource to evaluate progress in morbidity control. The aims are to identify communities in Ghana where the integrated distribution of praziquantel and albendazole could be prioritized to maximize the impact on morbidity and generate output maps which can constitute an evidence base to be used by GHS NTD program managers for the evaluation of ongoing interventions.

## Materials and Methods

### Ethics Statement

Ethical approval for these surveys was obtained from Imperial College Research Ethics Committee UK and the Ghana Health Service Ethical Review Committee in Ghana. The official letters were sent by the Ghana Health Service to the Regional and District health and educational authorities in advance. All data collection activities were carefully explained to, and oral consent was obtained from traditional authorities and other opinion leaders in the village (the village head, elders and political leaders), the school headmasters, the parent-teachers association, the representative of the pupils' parents and the local health authorities. All parents/guardians of all children involved in the study provided consent; parents/guardians who did not want their children to participate informed the school authorities. Child participants were given an explanation of the data collection activities and were free not to participate if they so chose. Written consent was not obtained and oral consent was approved by the ethics committee involved because the survey was considered by the UK and Ghanaian ethical committees as part of the monitoring and evaluation of routine health activities carried out by the GHS NTD control program. Representatives of parent-teacher associations were invited to be present during the sample collection process. Most of the parents who showed up at the school were there to ensure that their children had the opportunity to participate. They were also given the opportunity to have a look at microscope slides that had the parasite eggs present.

### Data

The parasitological data for this study were collated from national, school-based parasitological surveys conducted in Ghana in 2008 with the support from the SCI [9]. These surveys were originally designed and implemented with the objective of mapping urinary schistosomiasis, which is the most prevalent form of schistosomiasis in West Africa [22], [35]. As the geographical coordinates of the schools were not known, but the district and locality (rural or urban) of each school is known, a stratification procedure was used to select the schools such that schools in rural communities or districts that were adjacent to Lake Volta were twice as likely to be sampled as schools in urban communities or districts that were not adjacent to Lake Volta. Districts were stratified into 4 strata: a) those adjacent to Lake Volta (stratum 1); b) districts not adjacent to the lake (stratum 2); c) schools which are located in rural areas (stratum 3); and d) schools which are located in urban areas (stratum 4). The stratum that is adjacent to the lake (stratum 1) was sampled twice as densely as the stratum away from the lake (stratum 2) to ensure that accurate estimates of schistosomiasis are obtained. The rural stratum was sampled more densely than the urban stratum to ensure there were enough data for rural areas which are expected to have higher prevalence of both schistosomiasis and soil-transmitted helminths. To ensure good geographical coverage of the survey area the number of schools to be selected from each district was calculated proportional to the size of the district. The area of each district was calculated using a geographical information system (GIS).

The sample size was calculated to give the same spatial density of schools as for similar surveys from neighbouring West African countries also supported by SCI (i.e. Burkina Faso, Mali and Niger) [13], [14]. It was decided to survey 77 schools and select at random 60 children (30 boys and 30 girls) in each school when possible. The sampling frame for school selection consisted of a list of all schools in the country, stored in a Microsoft Excel 2007spreadsheet. Children were selected from within the selected schools using systematic random sampling of class lists. Selected children were assembled and asked to provide a stool and urine sample. A total of 4,577 children aged 2–19 year old were tested; these correspond to 43 fewer children than expected because fewer children were sampled in some schools; these schools were evenly distributed across the country. Stool samples provided by each child were used to make two slides which were examined microscopically using the semi-quantitative Kato-Katz technique for the eggs of STHs (*Ascaris lumbricoides*, *Trichuris trichiura* and hookworm) and *Schistosoma mansoni*. After collection of stool samples these were processed immediately and slides were prepared and examined in the field laboratory by experienced microscopists in diagnosing schistosomiasis and STHs, within 2 hours of preparation to increase detection of the more labile hookworm eggs, by the Kato-Katz thick smear technique using a 41.7 mg template [36]. The concentration of eggs was expressed as eggs per gram of faeces (epg). From urine samples, up to 10 mL were filtered through a polycarbonate membrane and the number of eggs of *S. haematobium* were counted and expressed as eggs per 10 mL of urine. The geographic location of the school was determined using a handheld global positioning system device. The dataset for analysis included data from 4,445 children aged 5–19 years located in 77 schools from which complete demographic and parasitological information was available. A summary of the prevalence of each parasite for the study area is presented in Table 1, showing that *T. trichiura* was a rare STH infection in the study area. The prevalence of *S. mansoni* was also very low in comparison to that of *S. haematobium*. In our analyses we considered the data for *S. haematobium* and hookworm only because multiple infections with these parasites are known to be associated with pronounced morbidity, including anaemia [21].

**Table 1:** Prevalence of infection and infection intensity profile for schistosomiasis and soil-transmitted helminthiasis in Ghana, 2008.

Parasite	Without infection (n; %)	Mean infection prevalence (95% CI)*	Mean infection intensity (95% CI)*	Light-intensity infections (n; %)	Moderate-intensity infections (n; %)	Heavy-intensity infections (n; %)
*S. haematobium* §	3,803; 83.09	16.91 (15.82, 18.00)	23.09 (18.67,27.51)	447; 57.8	none	327; 42.2
*S. mansoni* †	4,526; 98.89	1.11 (0.81, 1.42)	3.71 (1.94,5.47)	17; 33.3	25; 49.0	9; 17.7
Hookworms¥	4,397; 96.07	3.93 (3.37, 4.50)	4.44 (2.95,5.92)	180; 100.0	none	none
*A. lumbricoides* χ	4,439; 96.98	3.02 (2.52, 3.51)	9.36 (3.08,15.65)	135; 97.8	3; 0.1 2.2	none
*T. trichiura* ζ	4,556; 99.54	0.46 (0.26, 0.66)	0.36 (0.12,0.61)	21; 100	none	none

*the confidence intervals (CIs) account for clustered survey design. The total sample size is 4,577 children.

**§:** light infection <50 eggs/10 mL, heavy infection: >50 eggs/10 mL;

**†:** light infection: 1–99egs per gram of faeces (epg), moderate infection: 100–399epg, heavy infection: >400epg;

**¥:** light infection:1–1,999epg, moderate infection: 2,000–3,999epg, heavy infection: >4,000epg;

**χ:** light infection: 1–4,999epg; moderate infection: 5,000–49,999epg, heavy infection: >50,000;

**ζ:** light infection: 1–999epg, moderate infection: 1,000–9,999epg; heavy infection: >10,000epg (Source: [39]).

The survey data were summarized by prevalence of mono- and co-infections and arithmetic mean infection intensity, by survey location. These summary data were plotted in ArcGIS version 10 (ESRI, Inc). To provide robust confidence intervals around the mean prevalence in Ghana prevalence estimation took into account the clustered design of the sampling, using the school as a primary sampling unit and including adjustments for the probability of sampling and finite population corrections for sampling without replacement in the Stata/SE 11.0 statistical package (StataCorp, College Station, Texas, USA). This was based on the assumption that children attending the same school would be more likely to have more similar exposures than children attending other schools. Electronic data for land surface temperature (LST) and normalised difference vegetation index (NDVI) were obtained from the National Oceanographic and Atmospheric Administration's (NOAA) Advanced Very High Radiometer (AVHRR; see Hay et al. [37] for details on these datasets) and the location of large perennial inland water bodies was obtained from the Food and Agriculture Organization of the United Nations (http://www.fao.org/geonetwork/srv/en/main.home). Values for LST, NDVI and distance to the nearest perennial inland water body (PIWB) were extracted in ArcGIS version 10.0 (ESRI, Inc) for each survey location.

### Spatial Risk Model of Parasite Co-Infection

The initial set of variables included the individual-level variables of sex and age (categorized into 5–9, 10–14 years and 15–19 years) and the school-level variables of NDVI, LST and distance to PIWB. Fixed-effects multinomial regression models of *S. haematobium*/hookworm co-infections were developed in a frequentist statistical software package (Stata version 10.1, Stata Corporation, College Station, TX). A quadratic association between LST and prevalence was assessed and was not found to improve model fit (using Akaike's Information Criterion [38]); distance to PIWB was significantly and negatively associated with prevalence of co-infection. NDVI was not found to be significantly associated with prevalence of co-infection in the preliminary multivariable models and was excluded from further analysis (Wald's *P*>0.2). Therefore, it was decided to enter LST and distance to PIWB as covariates into the final spatial models in WinBUGS. In the MBG co-infection model, individual raw survey data were aggregated into groups according to age group, sex and location and using four infection outcomes (i.e. 1 = Without infection; 2 = *S. haematobium* mono-infection; 3 = hookworm mono-infection and 4 = *S. haematobium*-hookworm co-infection). In this model the baseline category was “Without infection”. Statistical notation of Bayesian geostatistical models is presented in Text S1.

### Spatial Risk Models of Parasite Infection Intensity

Individual egg count data were used as a proxy of worm burden in the models of infection intensity. Infection intensity can be modeled by transforming parasite egg counts into an ordinal or nominal categorical variable based on World Health Organization (WHO) cut-offs (not infected, light-intensity infection, moderate and high-intensity infection) [39] and using a multinomial distribution for the stratified intensity outcomes. However, the multinomial approach involves stratifying egg counts, leading to a loss of information whereas the Poisson or the negative binomial approach make full use of infection intensity data on a continuous scale (as measured by number of eggs found in both slides per individual) [40]. Usually, only a small proportion of the infected population excretes large numbers of parasite eggs. Therefore, infection intensity data typically contain many zero egg counts due to the aggregation of parasite distribution among hosts (also referred to as over dispersion) [41] and the presence of false negatives [42]. The large number of zero counts suggests the data are over dispersed relative to the Poisson distribution, the usual discrete probability distribution used for count data. To address this problem, the zero-inflated Poisson (ZIP) or the zero-inflated negative binomial (ZINB) regression models could be used [21], [43], [44]. The best fitting distributional form of parasite egg counts was investigated using the *nbvargr* command in Stata version 11 (Stata Corporation, College Station, TX); this assessment provided statistical support to consider the ZIP distribution as the best possible fit to the data for both parasites. We then developed univariate and multivariate models of parasite egg counts using ZIP models for each parasite species in Stata version 11. The variable screening approach was similar to that outlined above for models of co-infection and for *S. haematobium* and hookworm it was decided to enter untransformed LST and distance to PIWB as environmental covariates into the final spatial models in WinBUGS. We have applied a MBG ZIP model following an approach which is similar for prediction of prevalence, using the same candidate set of predictor variables and geostatistical random effects as the ones used in the co-infection model. The main difference was that the outcome, rather than being binary (infected/not infected), was a count [epg (for hookworm) or eggs per 10 mL of urine (for *S. haematobium*)] modeled using a Poisson distribution for the mean intensity models. With a ZIP model there are two processes that have to be considered, 1) the zero inflation model and 2) the (positive) expectation of the response for the distribution of the Poisson (or other distribution) part of the model. The marginal (or overall) expected value of the response is the expected value of the Poisson part shrunken by an amount proportional to the zero inflation probability [45]. Statistical notation of MGB ZIP models is presented in Text S2.

### Parameter Estimation, Spatial Prediction and Model Validation

The spatial models were fitted in WinBUGS version 1.4 statistical software (Medical Research Council Biostatistics Unit, Cambridge, United Kingdom and Imperial College London, United Kingdom) and were based on MBG [46]. For each model (i.e. single infection intensity models and the multinomial model of co-infection) a burn-in of 5,000 iterations was used followed by 5,000 iteration intervals after which convergence was assessed using visualization of history and density plots of the series of posterior values. Bayesian model outputs for parameters of interest and for predictions at unsampled locations are probability distributions, termed posterior distributions, which represent the probability of a variable of interest taking each of a range of plausible values. The posterior distributions can be summarized by statistics such as the posterior mean and 95% Bayesian credible interval (BCI). For model coefficients, significance at the 5% level is defined by a 95% BCI that excludes zero.

In all models, convergence of model parameters was successfully achieved after 20,000 iterations and the model was run for a further 10,000 iterations, after which the predicted prevalence for each outcome group at unsampled locations was stored for boys of 15–19 years of age. The models developed allow production of predictive maps of co-infection and infection intensity for all age groups and sexes – for mapping purposes we chose to map boys aged 15–19 years, as this was the group with the highest risk of co-infection and the highest hookworm intensities of infection. Predictions were made at the nodes of a 0.1×0.1 decimal degree grid (approximately 12 km^2^) by adding (on the logit scale) the following: 1) the sum of the products of the coefficients for the fixed effects and the values of the fixed effects at each prediction locations, and 2) the interpolated random effect. The latter was achieved using the *spatial.unipred* command in WinBUGS [47], which implements Bayesian kriging. This function implements independent simulations that do not consider neighboring values, as opposed to joint prediction which is conditional on the values of neighboring locations. While joint prediction yields more accurate measures of prediction uncertainty, it was not considered feasible in this study due to having extremely demanding computational requirements.

The area under the curve (AUC) of the receiver operating characteristic was used to determine discriminatory performance of the model predictions relative to observed co-infection prevalence thresholds of 5% and 10% [48]. Following the same procedure, the predicted infection intensity was compared to the observed intensity of infection, dichotomised at 50 eggs/10 mL, for *S. haematobium* and at 1 epg of stool for hookworm. An AUC value of 0.7 was taken to indicate acceptable predictive performance [48].

### Mapping Parasite Co-Intensity

The *S. haematobium* and hookworm co-intensity map for Ghana was constructed by overlaying the predicted posterior mean intensity maps of *S. haematobium* and hookworm in ArcGIS version 10.0 (ESRI, Inc.). WHO classifies infection intensity based on eggs count thresholds [39]: for *S. haematobium* light infections are 1–50 eggs/10 mL of urine and heavy infections are >50 eggs/10 mL of urine, and for hookworm light infection are 1–1,999epg, moderate infection was 2,000–3,999epg, heavy infection was >4,000epg. For mapping purposes the predicted intensity of *S. haematobium* was defined as <25 eggs/10 mL of urine, >25–50 eggs/10 mL of urine and >50 eggs/10 mL of urine. Due to the low mean hookworm infection intensity in Ghana, the predicted intensity of infection for this parasite was dichotomized according to <1 and ≥1 epg.

## Results

### Survey Results

**Figure 1:**
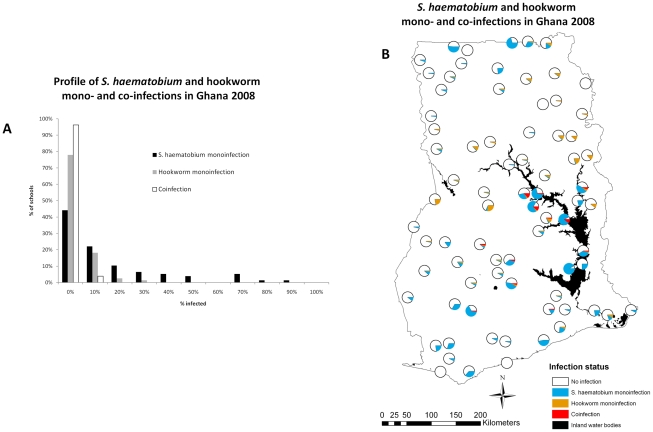
Observed *Schistosoma haematobium* and hookworm mono- and co-infections in children aged 5–19 years in Ghana, 2008. Data were collected prior to the inception of the Ghana Health Service control program for neglected tropical diseases.

The prevalence of *S. haematobium* and hookworm mono- and co-infection is presented stratified by sex and age (Table 2); our results show that males are significantly more co-infected than females and children of 15–19 years of age were significantly more co-infected than children of 5–9 years of age. The frequency distribution and spatial distribution of the raw prevalence of *S. haematobium* and hookworm mono- and co-infections for Ghana is presented in Figure 1. The bar chart in Figure 1A shows that the distribution of the school prevalence mono- and co-infection is markedly skewed; the map in Figure 1B shows that there is a distinct spatial heterogeneity of *S. haematobium* and hookworm co-infections in Ghana where most co-infections are distributed near the western bank of the Lake Volta in central Ghana.

**Table 2:** *Schistosoma haematobium* and hookworm mono- and co-infections in Ghana 2008, stratified by sex and age.

Infection status	Total	sex		*P* value*	age in years			*P* value*
		male	female		5–9	10–14	14–19	
Number of children	4,445	2,209	2,236	<0.001	483	3,045	917	<0.001
Without infection (%)	81.7	39.3	42.4		9.4	55.8	16.5	
*S. haematobium* mono-infection (%)	14.4	7.8	6.6		1.1	10.3	3.1	
Hookworm mono-infection (%)	3.2	2.1	1.1		0.4	2.0	0.8	
*S. haematobium* – Hookworm co-infection (%)	0.7	0.5	0.2		0.02	0.4	0.2	

*based on χ^2^-test of significance.

**Figure 2:**
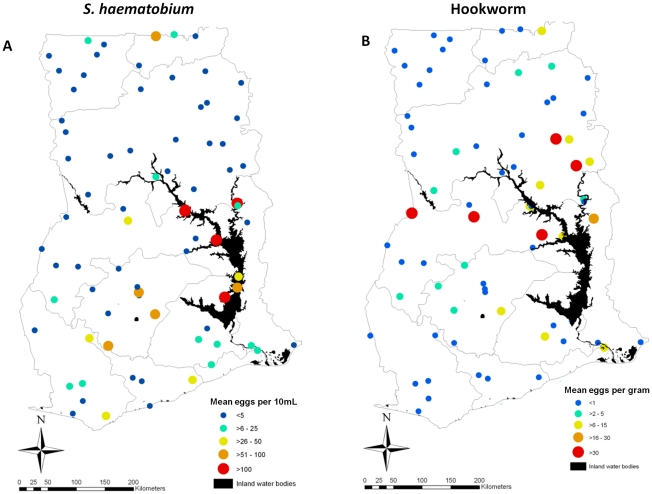
Spatial heterogeneity of observed *Schistosoma haematobium* and hookworm egg counts in children aged 5–19 years in Ghana, 2008.

Based on WHO classification guidelines [39], our results show that all hookworm infections in Ghana are of light intensity (1–1,999 epg) whereas 42% of the *S. haematobium* infections are of heavy intensity (>50 eggs/10 mL of urine) (Table 1). The spatial distribution of the raw intensity of *S. haematobium* and hookworm infections, as measured, respectively by the mean number of eggs per 10 mL of urine or epg in each location in 4,527 (for *S. haematobium*) and 4,538 (for hookworm) school children aged 5–19 at 77 locations in Ghana is presented in Figure 2. The map in Figure 2A suggests that *S. haematobium* heavy-intensity infections are distributed along the Lake Volta. Figure 2B suggests that the most intense hookworm infections are not localized around the Lake Volta but distributed across a wider area in central Ghana.

### Predicted Prevalence of *S. haematobium*/Hookworm Mono- and Co-Infections

Parameters in Table 3 represent the logarithm of the relative risk ratio of mono- and co-infections; inspection of the 95% BCI shows that males had a significantly higher prevalence of co-infection and mono-infections than females. In addition, the prevalence of *S. haematobium*-hookworm co-infections in children aged 15–19 years was significantly higher than in those of age 5–9 years. Furthermore, children aged 10–14 had significantly higher *S. haematobium* mono-infections than children aged 5–9 years. Distance to PIWB was significantly and negatively associated with *S. haematobium*-hookworm co-infections and hookworm mono-infection. The variable for LST was positively and significantly associated with hookworm mono-infections. Phi (*ϕ*) indicates the rate of spatial decay of spatial autocorrelation and varied from 50.3, 19.8 and 59.8 for *S. haematobium*-hookworm co-infection, *S. haematobium* mono-infection and hookworm mono-infections (Table 3). This indicates that, after accounting for the effect of covariates, the radii of the clusters were approximately 7 km, 18 km and 6 km for *S. haematobium*-hookworm co-infection, *S. haematobium* mono-infection and hookworm mono-infections (note, *ϕ* is measured in decimal degrees and 3/*ϕ* determines the cluster size; one decimal degree is approximately 111 km at the Equator). The tendency for spatial clustering was the weakest for hookworm mono-infections (the higher value the spatial variance parameter the higher the tendency for spatial clustering) (Table 3).

**Table 3:** Spatial effects for prevalence of *Schistosoma haematobium* and hookworm mono- and co-infections in Ghana, 2008.

Variable	*S. haematobium*-hookworm co-infectionPosterior mean (95% BCI)	*S. haematobium* mono-infectionPosterior mean (95% BCI)	Hookworm mono-infectionPosterior mean (95% BCI)
Male (versus female)	1.26 (0.41, 2.23)	0.36 (0.16, 0.54)	0.74 (0.37, 1.13)
Age 10–14 y (versus 5–9 y)	2.74 (−0.08, 7.86)	0.54 (0.12, 1.04)	0.28 (−0.36, 0.97)
Age 15–19 y (versus 5–9 y)	3.39 (0.46, 8.56)	0.43 (−0.04, 0.99)	0.43 (−0.27, 1.21)
Distance to PIWB*	−6.22 (−10.88, −2.83)	−0.11 (−0.62, 0.51)	−0.94 (−1.42, −0.53)
Land surface temperature*	−2.33 (−4.86, 0.09)	−0.33 (−0.91, 0.43)	0.62 (0.19, 1.12)
Intercept	−16.98 (−22.51, −10.27)	−3.38 (−3.99, −2.84)	−4.85 (−5.68, −4.05)
*ϕ* (rate of decay of spatial correlation)	50.29 (10.11, 96.78)	19.78 (1.94, 85.17)	59.80 (11.51, 98.19)
*σ* ^2^ (variance of spatial random effect)	17.45 (4.85, 47.52)	4.23 (2.67, 6.75)	1.77 (0.86, 3.26)

*Variables were standardized to have mean = 0 and standard deviation = 1; BCI = Bayesian credible interval; PIWB = perennial inland water body.

**Figure 3:**
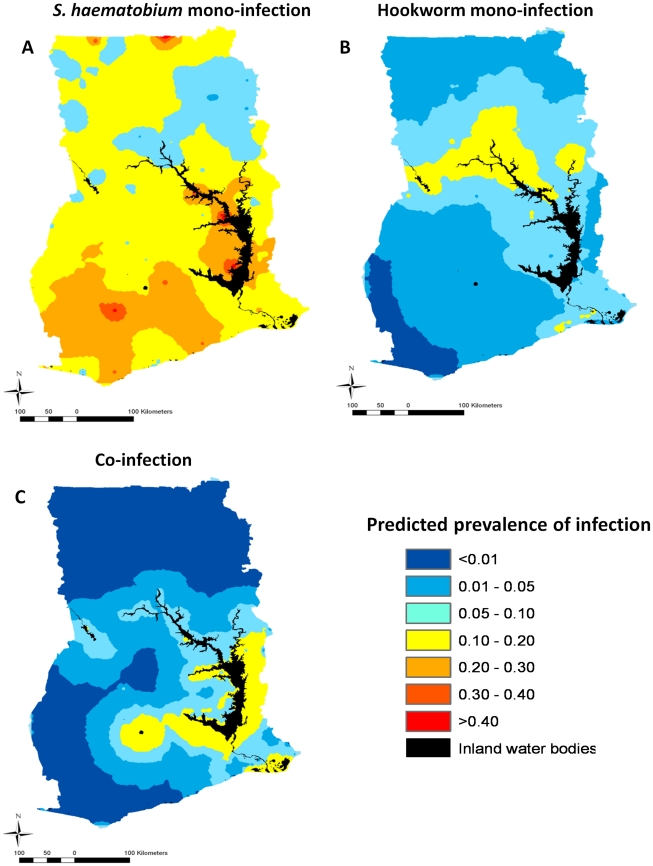
Predicted prevalence of *Schistosoma haematobium* and hookworm mono-and co-infections in boys aged 15–19 years in Ghana, 2008.

The geographical distribution of the risk of *S. haematobium* mono-infection (Figure 3A) is widespread and heterogeneous across Ghana, while the distribution of the risk of hookworm mono-infection (Figure 3B) is also geographically heterogeneous but much more focal. In Figure 3A, the risk of *S. haematobium* mono-infections is highest (>30%) in areas adjacent to the Lake Volta as well as in areas not associated with the Lake Volta in the south of the country. In contrast, the risk of hookworm mono-infection is highest (>5%) in areas in the eastern bank of the Lake Volta and areas located in a mid-latitudinal band across Ghana, not directly associated with the Lake Volta. In Figure 3C, the risk of *S. haematobium* and hookworm co-infections is quite focal and associated with areas adjacent to the Lake Volta and is highest (>5%) in the East Bank and in the South West of the lake. This model was able to predict with an AUC of 0.78 (0.70, 0.85) and 0.75 (0.68, 0.81) using a cut off of 5% and 10% prevalence, respectively.

### Predicted Intensity of *S. haematobium* and Hookworm Infections

**Figure 4:**
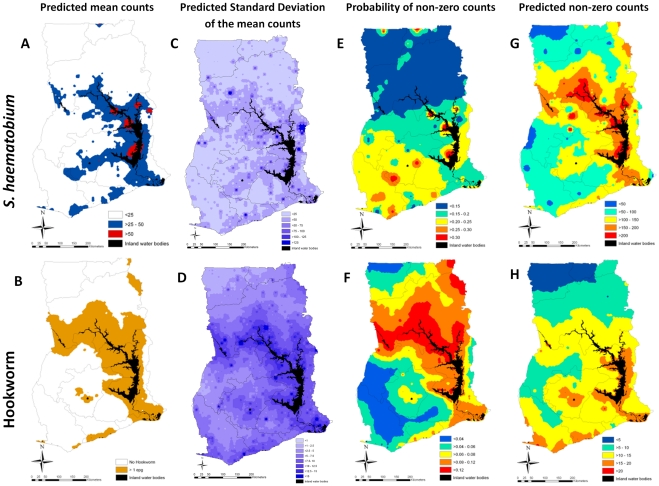
Mapped outputs for *Schistosoma haematobium* and hookworm egg count models, boys aged 15–19 years in Ghana, 2008. Egg counts for *S. haematobium* are as per 10 mL urine; egg counts for hookworm are as per gram of faeces.

Estimates presented in Figure 4 are the mean (marginal) posterior predicted intensity values (A and B), the standard deviation of the predicted mean egg counts (C and D), mean probability of intensity being non-zero (E and F), and the predicted mean non-zero counts (G and H), from Bayesian geostatistical models. Males and children aged 10–14 years had the highest intensity of *S. haematobium* infections, whereas children aged 15–19 years had the highest intensity of hookworm infections (Table 4). Distance to PIWB was negatively associated and LST was positively associated with *S. haematobium* infection intensity. None of the environmental variables were significantly associated with hookworm infection intensity. After accounting for the effect of covariates, intensity of infection was clustered with a radius of approximately 17 km and 6 km for *S. haematobium* and hookworm respectively. The tendency for spatial clustering was the strongest for hookworm infection intensity (Table 4). The models of *S. haematobium* and hookworm infection intensity were able to predict the geographical distribution of infection intensity with an AUC 0.82 (95% CI: 0.75, 0.88) and 0.78 (95% CI: 0.73, 0.85) using a cut-off of 50 eggs per 10 mL of urine and 1 epg, respectively.

**Table 4:** Spatial effects for intensity of *S. haematobium* and hookworm infections in Ghana, 2008.

Variable	*Schistosoma haematobium*Posterior mean(95% BCI)	HookwormPosterior mean(95% BCI)
Male (versus female)	0.08 (0.06, 0.09)	0.46 (0.33, 0.60)
Age 10–14 y (versus 5–9 y)	0.41 (0.39, 0.44)	0.76 (0.54,1.00)
Age 15–19 y (versus 5–9 y)	0.13 (0.10, 0.16)	1.10 (0.84, 1.37)
Distance to PIWB*	−0.48 (−0.68, −0.28)	−0.45 (−0.88, 0.08)
Land surface temperature*	0.26 (0.12, 0.47)	−0.11 (−0.57, 0.33)
Mean zero dispersion	0.83 (0.82, 0.84)	0.96 (0.96, 0.97)
Intercept	2.68 (2.48, 2.94)	−0.09 (−0.55, 0.30)
*ϕ* (rate of decay of spatial correlation)	21.23 (5.36,61.80)	56.56 (13.19, 140.90)
*σ* ^2^ (variance of spatial random effect)	3.47 (2.60, 4.63)	1.52 (0.98, 2.26)

*Variables were standardized to have mean = 0 and standard deviation = 1; BCI = Bayesian credible interval; PIWB = perennial inland water body.

### Mapping of *S. haematobium* and Hookworm Co-Intensity

**Figure 5:**
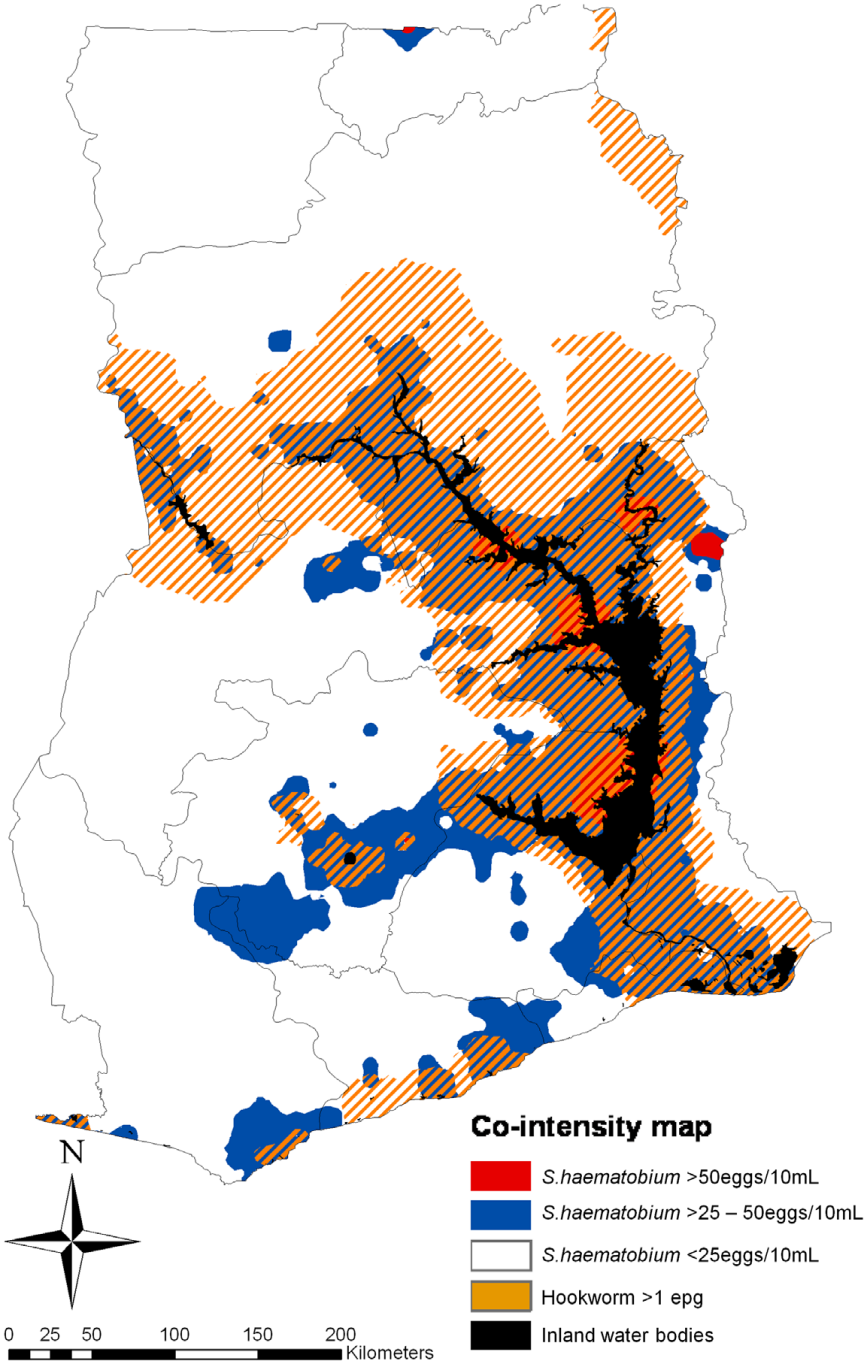
Predicted areas of co-intensity for *Schistosoma haematobium* and hookworm, boys aged 15–19 years in Ghana, 2008.

A map showing the geographical distribution of the mean co-intensity profile for boys aged 15–19 years in Ghana is shown in Figure 5. This map indicates that the areas where high intensity *S. haematobium* infection co-exists with areas where intensity of hookworm infection was predicted to be ≥1 eggs per gram are localised to small areas adjacent to the Lake Volta. These areas are surrounded by areas where light to moderate *S. haematobium* infections co-exist with hookworm infections of ≥1 epg. Visual inspection of Figure 3C and Figure 5 suggests that the risk of *S. haematobium* and hookworm co-infections is highest (>10–20%) in areas where *S. haematobium* infections co-exist with hookworm infections of ≥1 epg. In addition, the area in the eastern bank of the Lake Volta where the highest prevalence of co-infection was predicted (Figure 3C) coincides with an area where *S. haematobium* and hookworm co-intensity is predicted to be >25–50 eggs/10 mL and ≥1 epg. However, the area where co-infection is >15% is located in a different area where co-intensity is predicted to be highest (50 eggs/10 mL and ≥1 epg).

## Discussion

We have investigated the geographical risk of *S. haematobium* and hookworm co-infection, predicted the intensity of *S. haematobium* and hookworm infections (as measured by individual egg counts) and presented a novel application of Bayesian geostatistical modeling to predict the geographical location of areas where the highest intensity of infection of *S. haematobium* and hookworm co-exist. For that, we have used contemporaneous and robust statistical modeling methods on recent, extensive and representative infection data from Ghana. Results identify the importance of environmental risk factors in explaining national geographical variation of the prevalence of mono- and co-infection and intensity of infection with these parasites. This study also shows the potential value of combining co-infection and co-intensity maps when the focus of parasitic disease control planning is geographically heterogeneous and when a sensitive benchmark for control evaluation is required.

### The Burden of Helminth Co-Infections and the Distribution of Co-Intensity in Ghana

All existing studies of the spatial epidemiology of mono- and co-infection focus on *S. mansoni* and hookworm and highlight the marked spatial heterogeneity in patterns of infection [3], [19], [49]. Our modeling shows that it is also possible to predict spatial patterns of *S. haematobium*–hookworm co-infection at the national scale in Ghana. The observed risk factors (i.e. distance to water bodies and land surface temperature) are already well established and are consistent with the known epidemiology of *S. haematobium* and hookworm infection [3].We demonstrated that the distribution of hookworm mono- and co-infection in Ghana is highly focal, exhibiting a highly skewed frequency distribution and a marked spatial dependency. In contrast, the distribution of *S. haematobium* is geographically heterogeneous and is more widespread. The generally similar patterns of hookworm mono- and co-infection suggest that the localized spatial distribution of co-infection in Ghana is influenced by the distribution of hookworm, rather than the distribution of *S. haematobium*. This finding is not consistent with those from East African countries where the geographical distribution of co-infection was found to be generally influenced by the distribution of *S. mansoni* rather than hookworm [3]. This reflects the fact that, in East Africa, schistosomiasis is focal and hookworm is ubiquitous, whereas in West Africa, schistosomiasis is more widely distributed and hookworm is relatively rare. The relative focality and low level of hookworm infection in Ghana may be driven by the MDA of anthelmintics for the control of other parasitic infections. The Global Program to Eliminate Lymphatic Filariasis (GPELF) distributes ivermectin and albendazole for LF across the region [24]–[28] and it is possible that the administration of albendazole for LF control in parts of Ghana may have had an important impact on STH prevalence and associated geographical distribution. The transmission dynamics of hookworm depends on microclimatic suitability for infective larvae survival (primarily temperature and humidity) and exposure opportunities to environments contaminated with human excreta. However, in our analyses we did not find statistical support for the inclusion of remotely sensed rainfall data (as measured by NDVI) as an environmental covariate in any of our models. It is possible that the effect of these programs have confounded substantially the relationship between environmental determinants known to influence the geographical distribution of hookworm.

We have also predicted for Ghana the spatial distribution of *S. haematobium* and hookworm infection intensity. While the predictive infection intensity for hookworm was low across most of the country, our approach generated local estimates of infection intensity for both parasites that highlighted the role of geographical heterogeneity in intensity of infection on parasite co-infection profiles. When combining the predictive intensity of infection maps for *S. haematobium* and hookworm, we found similarities between the distribution of co-infections (Figure 3C) and co-intensity (Figure 5), which is consistent with recent evidence suggesting that multiple helminth infections tend to cluster more with increasing levels of intensity of transmission [5], [6]. However, there were subtle differences that suggest it might be worth using both co-infection and co-intensity mapping approaches when planning integrated control programs over those that use crude prevalence or intensity maps alone.

### Using Helminth Co-Infection and Co-Intensity Maps to Monitor and Evaluate Control Efforts

Considering that intensity of infection is both an important epidemiological driver of morbidity and very sensitive to intervention efforts, the maximal effect of integrated morbidity control and evaluation could be achieved by geographically targeting areas where *S. haematobium* intensity co-exist with areas of hookworm intensity. In addition, targeting these areas would provide adequate transmission control by contributing to the reduction of the level of environmental contamination. While an empirical map of co-infection would allow the enumeration of population that are in need of treatment for multiple infections, its combined use with a co-intensity map constitutes an important cartographic resource by allowing the evaluation of the impact of control programs with the aim of reducing population-level morbidity [17]. This could be objectively achieved by conducting follow up surveys targeted to areas predicted to have the highest combined co-infection/co-intensity and assessing the degree of spatial contraction (or expansion) in the co-intensity surface following MDA.

Important uncertainties should be noted from the Ghanaian dataset and the predictions surfaces for parasite infection used in our models, which are likely to be propagated throughout the modeling framework. First, we used threshold egg counts to classify light, moderate and heavy intensity infections for each species [39]. In the case of hookworm it has been shown that the relationship between worm burden and egg output is non-linear, i.e. density-dependent, and differing between communities [50]. These non-linear phenomena influence the validity of infection intensity as measured by egg concentration in urine or in faeces and detection of parasite eggs simply indicates the presence of at least one sexually mature and mated female worm. However, egg counts for hookworm infections were consistently low and for that reason these may actually reflect low adult parasite burdens although heavy infections of asexual female or male worms may also be possible. Furthermore, the egg counts were based on a single sample which limits the Kato-Katz test performance for detecting low infection intensities such as the ones identified in our surveys [51]. Stool processing times greater than 30 minutes are also likely to result in low hookworm intensities being detected due to egg lysis. While the effect of the latter was minimized by prompt field processing of samples, the former is likely to be a limitation of the study as it may lead to greater variability of the estimates of infection intensity and therefore less precision in the mean prediction estimates shown in our maps.

Second, all hookworm infections were of low intensity and for that reason we chose to categorize our intensity map for hookworm into <1 epg and ≥1 epg for the generation of the co-intensity map outputs. Whilst the data used are a pre-intervention dataset, the degree to which the observed level of hookworm infection would be obscured by ongoing, small scale and spatially variable interventions efforts as mentioned above is difficult to quantify (there was some LF control with anthelmintics prior to 2008). However, based on the stratified design of the sampling protocol, we think that the data collected should be a good representation of both schistosomiasis and STHs before the GHS NTD control program was implemented, although some bias may be present in districts not adjacent to Lake Volta as some high hookworm transmission areas may have been missed. While the stratified approach to sampling adopted in our survey is adequate to estimate prevalence of schistosome infections and co-infections, future work should consider balanced spatial sampling schemes which account for geographical differences in other helminth species distributions. The surveys targeted children 5–19 years but the age-intensity profile for both helminth infections are different, with maximum intensity occurring at 10–14 years for schistosomiasis and 20–25 years for hookworm [52], [53]. In our modeling approach we found statistical support to generate co-infection and co-intensity maps for the 15–19 age group. While this may have been adequate for *S. haematobium* infection, it may not have been the case for hookworm, and predictive surfaces for this parasite are likely to represent under estimates. However data for the older age groups (i.e. 20–25 year of age) were not available. Nevertheless, given that low-intensity infections are not trivial and have been shown to cause significant morbidity, particularly when occurring as co-infections with other parasites [54], [55], the resulting predictive co-intensity map when combined with the co-infection map represents a rich source of information for decision makers with the aim of integrated morbidity control in Ghana.

### Potential Geostatistical Improvements

A number of potential improvements to the geostatistical approach to modeling co-infection and co-intensity could be adopted in the following ways. First, it has been shown that the diagnostic sensitivity of a single Kato-Katz thick smear or urine slide examination is low due to significant day-to-day and intra-specimen variation [42] and low infection intensities are likely to be missed unless multiple samples over consecutive days are collected [56], [57]. The predictive ability of our co-infection and intensity models could be improved in future iterations of these maps by modeling diagnostic uncertainty within the MBG framework [58].

Second, the fact that parasite infections and co-infections occur at particular locations in Ghana may partly be due to unmeasured covariates, such as poverty indicators (e.g. socio-economic status, access to clean water and sanitation). While a quarter of the variability in multiple-parasite associations can be explained by factors associated with the domestic environment, environmental factors have been shown to have an important role in driving these associations [59]. However, for remotely-sensed environmental factors included in our models the mean value was used as a proxy for the true environmental exposure distribution of pre-school children included in the analysis. This approach provides a somewhat imprecise measurement of exposure and therefore may result in regression dilution bias arising from imprecise exposure measurement which is most likely to lead to underestimation of the observed environmental effects [60]. Our ecological modeling approach could also be improved by including socio-economic status (a well known risk factor for infection at small spatial scales [19]) as a contextual covariate but a high-resolution poverty map for the study area was not available. Alternatively, a better understanding of sub-national variation in co-infection and co-intensity could be achieved in future iterations of our maps by adopting an individual level modeling approach and extending our models to include factors associated with the domestic environment of each child.

Finally, although the combined use of the resulting co-infection and co-intensity maps allows delineating areas where highest morbidity could be present, it does not allow estimating the number coinfected with high infection intensity profiles [17]. This feature would be important for resource planning and allocation. This could be achieved by categorizing species infection profiles (e.g. *S. haematobium*; hookworm) by intensity of infection (high, moderate, low; or by its percentiles) and combining for each individual the parasite-intensity for one parasite with parasite-intensity with the other parasite. In doing so, the present model could be extended to its multivariate analogue taking into account a multivariate spatial process [61]. The resulting maps would then allow evaluation of the geographical variation of multiple infections of differing intensities, including the estimation of the number of individuals co-infected with different intensities.

### Conclusions

The combination of co-infection and co-intensity maps allows the identification of sub-groups of the population which play an important role in environmental contamination (due to high egg ouput) and are at increased risk of severe morbidity (due to multiple species, heavy intensity parasite infections [54], [62]). The maps produced by our approach could be used by national program managers as decision-support tools for targeting the geographical delivery of integrated MDA to areas where intense transmission may be occurring and evaluate the progress of the national program. In the future, these maps could be updated in subsequent methodological iterations to incorporate further modeling refinements.

## Supporting Information

Text S1 Statistical notation of Bayesian geostatistical models for prevalence of *Schistosoma haematobium/hookworm* co-infection in Ghana, 2008.
(DOC)

Text S2 Statistical notation of Bayesian geostatistical models for infection intensity of *Schistosoma haematobium* and hookworm in Ghana, 2008.
(DOC)

Checklist S1 STROBE checklist.
(DOC)
