# Supplementary material for: Mapping Helminth Co-Infection and Co-Intensity: Geostatistical Prediction in Ghana
Source: PLoS Negl Trop Dis. 2011 Jun 7;5(6):e1200. doi: 10.1371/journal.pntd.0001200 (PMC3110174; doi:10.1371/journal.pntd.0001200)
Supplement: Text S1 — Statistical notation of Bayesian geostatistical models for prevalence of Schistosoma haematobium/hookworm co-infection in Ghana, 2008. (DOC) [file pntd.0001200.s001.doc]

**Text S1. Statistical notation of Bayesian geostatistical models for prevalence of *Schistosoma haematobium/Hookworm co-infection* in Ghana 2008.**

The MGB model for co-infection was of the form of:

$Y{}_{ijk}\sim Multinomial(p_{ijk},n_{ijk})$, and

$$p_{ijk}=\frac{\varphi_{ijk}}{\sum_{k} \varphi_{ijk}}$$

where *Y_ijk_* is the number of infection positive children in school *i*, age-sex group *j* and outcome group *k*, *n_ijk_* is the number of children examined, *p_ijk_* is the probability of infection. The reference value of 1 was given to the no-infection group and, therefore, $\varphi_{ijk}$is the odds of being in a specific outcome group relative to the no-infection group. For the remainder three outcome groups we fitted the nominal regression equations

$$logit(\varphi_{ij2})=\alpha_{2}+\sum_{z=1}^{Z} \beta_{z2}\times x_{zij}+u_{i2}$$

$$logit(\varphi_{ij3})=\alpha_{3}+\sum_{z=1}^{Z} \beta_{z3}\times x_{zij}+u_{i3}$$

$$logit(\varphi_{ij4})=\alpha_{4}+\sum_{z=1}^{Z} \beta_{z4}\times x_{zij}+u_{i4}$$

where *α_k_* is the outcome group specific intercept, *β* is a matrix of Z coefficients and *x* is a matrix of Z covariates, and *u_i_* are coefficients representing a geostatistical random effects. These random effects have a multinomial normal distribution, of mean zero and variance-covariance matrices defined by an isotropic powered exponential spatial correlation function:

$f(d_{ab};\varphi)=\exp\left[-(\varphi d_{ab})\right]$,

where *d_ab_* are the distances between pairs of points *a* and *b*, and $\varphi$ is the rate of decline of spatial correlation per unit of distance. Non-informative priors were used for *α* (uniform prior with bounds -$\infty$ and $\infty$) and the coefficients (normal prior with mean = 0 and precision = 1 × 10^-4^). The prior distribution of $\varphi$ was also uniform with upper and lower bounds set at 0.1 and 50. (the lower bound set to ensure spatial correlation at the maximum separating distance between survey locations was <0.5, assisting identifiability [1]).The precision of *u_i_* was given a non-informative gamma distribution.

**References**

1. Thomas; A, Best; N, Lunn; D, Arnold; R, Spiegelhalter D (2004) GeoBUGS User Manual. Cambridge: Medical Research Council Biostatistics Unit.
