# Supplementary material for: Mapping Helminth Co-Infection and Co-Intensity: Geostatistical Prediction in Ghana
Source: PLoS Negl Trop Dis. 2011 Jun 7;5(6):e1200. doi: 10.1371/journal.pntd.0001200 (PMC3110174; doi:10.1371/journal.pntd.0001200)
Supplement: Text S2 — Statistical notation of Bayesian geostatistical models for infection intensity of Schistosoma haematobium and hookworm in Ghana, 2008. (DOC) [file pntd.0001200.s002.doc]

**Text S2. Statistical notation of Bayesian geostatistical models for infection intensity of *S. haematobium* and hookworm in Ghana 2008.**

The MGB models for infection intensity were of the form of:

$Count{}_{i,j}\sim Poisson(n_{i,j})$,and

$$n_{i,j}=p_{i,j}*c_{i,j}$$

$$logit(p_{i,j})=\alpha+\sum_{z=1}^{Z} \beta_{k}\times x_{i,j,k}+u_{i}$$

$$log(c_{i,j})=\alpha+\sum_{z=1}^{Z} \beta_{k}\times x_{i,j,k}+u_{i}$$

*Where* $p_{i,j}$and $c_{i,j}$are the probability of non-zero infection intensity and the mean count of parasite eggs without taking overdisperstion into account in school *i*, age-sex group *j*, *α* is the intercept, *β* is a matrix of Z coefficients and *x* is a matrix of Z covariates, and *u_i_* are coefficients representing a geostatistical random effects. These random effects have a multinomial normal distribution, specified the same way as with models in Text S1. Non-informative priors were used for *α* (uniform prior with bounds -$\infty$ and $\infty$) and the coefficients (normal prior with mean = 0 and precision = 1 × 10^-4^). The prior distribution of $\varphi$ was also uniform with upper and lower bounds set at 0.1 and 50. (the lower bound set to ensure spatial correlation at the maximum separating distance between survey locations was <0.5, assisting identifiability [1]).The precision of *u_i_*, was given a non-informative gamma distribution.

**References**

1. Thomas; A, Best; N, Lunn; D, Arnold; R, Spiegelhalter D (2004) GeoBUGS User Manual. Cambridge: Medical Research Council Biostatistics Unit.
